# Characterization of Chitosan Persian Gum Hydrogel Containing Jaft and Yarrow Extracts for Enhanced Wound Healing

**DOI:** 10.1155/bmri/5592896

**Published:** 2025-08-27

**Authors:** Parisa Ahmadi, Maryam Jalili Tabaii, Zahra Kanannejad, Milad Mohkam

**Affiliations:** ^1^ Department of Microbiology, Faculty of Biological Sciences and Technology, Shahid Ashrafi Esfahani University, Isfahan, Iran, ashrafi.ac.ir; ^2^ Allergy Research Center, Shiraz University of Medical Sciences, Shiraz, Iran, sums.ac.ir

**Keywords:** antimicrobial activity, chitosan, hydrogel, jaft extract, Persian gum, wound healing, yarrow extract

## Abstract

This study presents the development and evaluation of a novel chitosan/Persian gum hydrogel loaded with an internal layer of oak fruit (Jaft) and yarrow extracts, designed to enhance wound healing. The hydrogel was synthesized using chitosan and Persian gum, selected for their biocompatibility and ability to promote wound healing. The incorporation of Jaft and yarrow extracts was aimed at augmenting the hydrogel’s therapeutic efficacy. Characterization of the hydrogel using Fourier transform infrared spectroscopy (FTIR), X‐ray diffraction (XRD), and scanning electron microscopy (SEM) confirmed the successful integration of the extracts and revealed a semicrystalline structure with a porous morphology conducive to controlled drug release. In vitro studies demonstrated a pH‐responsive release profile, with faster drug release at alkaline pH (8.5) compared to acidic and neutral pH (5.5 and 7.4). The hydrogel exhibited significant antibacterial activity, particularly against *Escherichia coli*, with minimum inhibitory concentration (MIC) and minimum bactericidal concentration (MBC) values lower than those observed for *Staphylococcus aureus*. Animal studies further validated the hydrogel’s efficacy, showing enhanced wound closure, increased epidermal thickness, higher fibroblast proliferation, and reduced infection rates compared to controls. These findings suggest that the chitosan/Persian gum hydrogel, enriched with natural extracts, holds significant potential as a multifunctional wound dressing, offering a synergistic approach to infection control and tissue regeneration.

## 1. Introduction

Wound healing is a complex and dynamic physiological process that the body initiates in response to injury [1]. This process comprises four distinct, yet overlapping phases: hemostasis, inflammation, proliferation, and remodeling. Initially, hemostasis involves the rapid cessation of bleeding through blood clot formation [2–5]. This is followed by the inflammatory phase, where immune cells such as neutrophils and macrophages are recruited to the wound site to eliminate debris and prevent infection, a process that typically lasts from 24 h to several days. The extracellular matrix (ECM), alongside stromal cells such as fibroblasts and myofibroblasts, plays a crucial role in supporting this repair process by providing structural integrity [6]. The proliferation phase, characterized by the formation of granulation tissue, includes the synthesis of new ECM components and the generation of epithelial cells, which collectively contribute to wound closure. Angiogenesis, or the formation of new blood vessels, also occurs during this phase, ensuring an adequate supply of oxygen and nutrients to the regenerating tissue. Finally, the remodeling phase restores the normal dermal structure and enhances the tensile strength of the healed tissue [7, 8].

Given the complexity of wound healing, the selection of appropriate wound dressings is critical for effective management [5, 9–11]. Ideal wound dressings should promote rapid healing, minimize discomfort, adhere well to damaged tissue, maintain a balanced moisture level, allow for oxygen exchange, and protect against infections [12]. Over the years, extensive research has focused on developing optimal materials to support wound healing and create a conducive environment for tissue repair [5]. Natural polymers have emerged as key players in this field, acting as accelerators of the healing process, reducing inflammation, and serving as safe, biocompatible frameworks for skin tissue regeneration [13, 14]. Among these, chitosan, a polysaccharide derived from crustacean shells, is particularly noteworthy for its bioactive properties that align with key criteria for rapid wound healing [15, 16]. Chitosan’s molecular structure closely resembles that of hyaluronic acid, a component of the skin’s ECM, enabling it to enhance cell adhesion, proliferation, and tissue regeneration [17, 18].

In addition to chitosan, Persian gum, an exudate polysaccharide derived from the wild almond tree (*Amygdalus scoparia*), has attracted attention for its biocompatibility, biodegradability, and ability to stimulate cell proliferation, making it a promising complementary agent to chitosan in wound healing applications [19, 20]. Recent studies have demonstrated that Persian gum can accelerate wound closure and increase the expression of genes involved in skin regeneration, leading to the development of sophisticated wound dressings and scaffolds [21].

Regarding the exploration of natural biomaterials, this study developed a novel chitosan/Persian gum hydrogel infused with Jaft and yarrow extracts. These plants were selected based on their traditional use and documented pharmacological activities relevant to wound healing. Yarrow (*Achillea millefolium*) contains bioactive compounds such as flavonoids, alkaloids, and sesquiterpene lactones, which exhibit synergistic antimicrobial and anti‐inflammatory activities by inhibiting microbial growth and modulating inflammatory mediators [22, 23]. Similarly, Jaft, the inner layer of oak (*Quercus brantii*) fruit, is rich in tannins, flavonoids, and phenolic acids known to possess strong antimicrobial properties through mechanisms like microbial cell wall disruption and enzyme inhibition [24–26]. The incorporation of both extracts aimed to harness their combined antimicrobial, anti‐inflammatory, and potentially proregenerative effects synergistically within the hydrogel dressing.

The primary objective of this study was to present a novel chitosan/Persian gum hydrogel infused with Jaft and yarrow extracts, designed to enhance wound healing through a multifaceted approach. In this investigation, we thoroughly examined the hydrogel’s surface characteristics, antibacterial activities, and morphology, as well as its healing efficacy in animal models.

## 2. Materials and Methods

### 2.1. Materials

Chitosan (Sigma‐Aldrich, medium MW, Germany), Persian gum purchased from the local market, Shiraz, Iran, glacial acetic acid (Merck, Germany), glutaraldehyde (Sigma‐Aldrich, Germany), ethanol (Merck, Germany), Jaft (*Quercus brantii* inner fruit layer), and yarrow (*Achillea millefolium*) were purchased from the local market, Shiraz, Iran, and authenticated. Nutrient broth (Himedia, India), Mueller–Hinton agar (Himedia, India), phosphate‐buffered saline (PBS) (Merck, Germany), ketamine (Bremer Pharma, Germany), xylazine (Bimeda, United States), hematoxylin (Merck, Germany), eosin (Merck, Germany), and all other reagents were of analytical grade.

### 2.2. Preparation of Yarrow and Jaft Extracts

In order to make yarrow (*Achillea millefolium*) and internal layer of oak fruit (Jaft) extracts, clean fresh plant materials were purchased from the local market in Shiraz, Iran. The plants were then identified and authenticated by a botanist at the Department of Pharmacognosy, Shiraz University of Medical Sciences, Shiraz, Iran. For yarrow, 20 g of chopped leaves and flowers was placed in a glass jar and added 200 mL of 70% ethanol to cover the plant completely. The jar was sealed and left to infuse at room temperature for 48 h. Every 12 h, it was gently shaken to help the bioactive compounds come out more. Once the soaking time was over, the mixture was put through a fine mesh strainer or cheesecloth to separate the solids from the liquid extract. The similar procedure was carried out for the preparation of Jaft extract. Twenty grams of chopped Jaft was mixed with 200 mL of 70% ethanol, sealed, and shaken for 48 h before they were strained. After the liquid extracts are made, they were freeze‐dried to keep the bioactive chemicals they contain. After being placed on freeze‐drying trays, the extracts were frozen at ‐40°C. After they have frozen, they were put in a freeze dryer where the pressure was reduced to about 0.01 mbar. This lets the ice turn into vapor without going through the liquid phase first. During the primary drying process, gradual heating was used to get rid of about 95% of the water content. Any remaining bound water was then removed during secondary drying, bringing the final moisture content down to about 1%–5%. After being freeze‐dried, the extracts were kept in dark glass cases to keep them safe from light and degradation for further experiments [27].

### 2.3. Chitosan/Persian Gum Hydrogel Preparation

**Figure 1:**
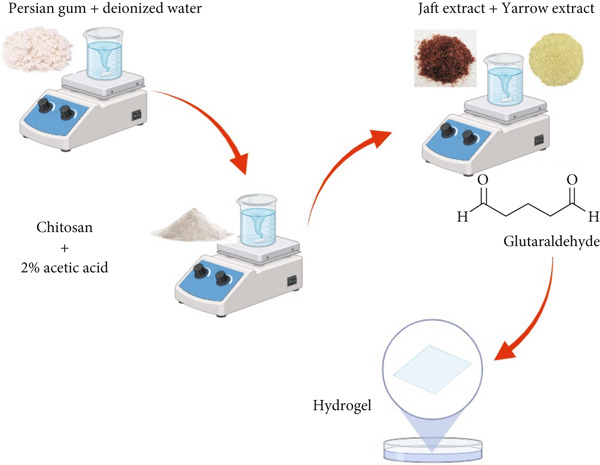
Schematic illustration of chitosan/Persian gum hydrogel synthesis process loaded with yarrow and Jaft extract.

In order to make the chitosan/Persian gum hydrogel, the chitosan was initially dissolved in a 2% acetic acid solution to form a uniform solution (Figure 1). Simultaneously, Persian gum was produced by dissolving a suitable quantity in deionized water to obtain a concentration of 2% (*w*/*v*). After preparing both solutions, the Persian gum solution was added slowly to the chitosan solution while stirring constantly to achieve thorough mixing. The resulting mixture was agitated for a further 15 min to facilitate the creation of the hydrogel network. Subsequently, the concoction was put into molds and let to solidify at ambient temperature for a duration of 24 h. The ultimate outcome was preserved in a desiccator to uphold its characteristics until subsequent utilization [28].

### 2.4. Loading Extracts to Chitosan/Persian Gum Hydrogel

To load yarrow and Jaft extracts into the chitosan/Persian gum hydrogel, 5 mL of each extract was mixed with the chitosan/Persian gum hydrogel solution prior to the gelation process. In this context, the extract solutions were added to the hydrogel mixture while stirring gently to ensure homogeneity and prevent the formation of bubbles. Following this, a cross‐linking agent (glutaraldehyde) was incorporated into the mixture at a concentration of 0.5% (*w*/*v*) to enhance the mechanical properties and stability of the hydrogel. The combined mixture was then poured into molds and allowed to gel at room temperature for 24 h and then stored at 4°C until later utilization [28].

### 2.5. Characterization of the Synthesized Hydrogel

Fourier transform infrared spectroscopy (FTIR), X‐ray diffraction (XRD), and scanning electron microscopy (SEM) investigations were performed to characterize the chitosan/Persian gum hydrogel that was loaded with yarrow and Jaft extracts. To conduct FTIR analysis, a sample weighing approximately 15 mg of the hydrogel filled with plant extracts was coarsely pulverized and then placed in a sample container. The spectra were obtained using a Tensor 25 series FTIR spectrometer (Germany) in a wavelength range of 4000–500 cm^−1^ in order to identify functional groups and evaluate the interactions between the components in the hydrogel matrix. The hydrogel’s crystalline structure was assessed using XRD analysis to identify any alterations in crystallinity resulting from the inclusion of the extracts. The samples underwent analysis using a PAN analytical diffractometer (X’pert Pro, United Kingdom) equipped with copper (Cu) radiation at a voltage of 40 kV. The scanning was performed over a 2*θ* range of 10°–100° at a rate of 2°/min. Ultimately, SEM was employed to examine the physical characteristics and microscopic arrangement of the hydrogel’s surface. Prior to imaging (VEGA 3, TE SCAN SEM, Czech Republic), hydrogel samples underwent gold‐coating to improve conductivity [29].

### 2.6. In Vitro Drug Release Study of Synthesized Hydrogel

To investigate the in vitro extract release profile of the synthesized chitosan/Persian gum hydrogel loaded with yarrow and Jaft extracts, a dialysis method was employed. The hydrogel samples, approximately 1 cm^3^ in size, are placed in a dialysis bag (MWCO: 12–14 kDa) with a molecular weight cutoff suitable for retaining the hydrogel matrix while allowing the release of the bioactive compounds. The dialysis bag was then submerged in PBS at pH 7.4 (as a release medium) to simulate physiological conditions. The release medium was maintained at a constant temperature of 37°C using a water bath to mimic body temperature. At predetermined time intervals (1, 2, 4, 6, 12, and 24 h), 5 mL of the release medium is withdrawn and replaced with an equal volume of fresh PBS to maintain sink conditions. The collected samples were analyzed using a UV‐visible spectrophotometer (U.VT60U; PG Instrument, England) at a wavelength of 380 nm to quantify the concentration of the released bioactive compounds from the hydrogel. The cumulative release data were plotted against time, and the release kinetics were evaluated by fitting the data to first‐order models to elucidate the release mechanism. Prior to the release assays, UV–vis absorption spectra of Jaft and yarrow extracts in PBS (pH 7.4) were recorded from 200 to 800 nm. Both extracts displayed pronounced absorption peaks at 379–382 nm; accordingly, 380 nm was chosen for all quantitative measurements. Calibration curves for each extract at this wavelength were generated (*R* ^2^ > 0.995), and method sensitivity (LOD, LOQ) was confirmed to be sufficient to detect cumulative release as low as 2% of the loading dose. Release samples (5 mL) withdrawn at predetermined intervals were analyzed at 380 nm (U.VT60U; PG Instrument, England), ensuring consistency and comparability across both extracts [30].

### 2.7. Antibacterial Activity Assay

To evaluate the antibacterial activity of the chitosan/Persian gum hydrogel loaded with yarrow and Jaft extracts, a disk diffusion method was employed against *Escherichia coli* (*E. coli*) and *Staphylococcus aureus* (*S. aureus*). Bacterial cultures were grown in nutrient broth at 37°C until they reached the logarithmic phase. The bacterial suspensions were then adjusted to a 0.5 McFarland standard, equivalent to approximately 1.5 × 10^8^ CFU/mL. Mueller–Hinton agar plates were inoculated with the adjusted bacterial suspensions using sterile cotton swabs, ensuring even distribution of the inoculum on the plates. Hydrogel samples, both loaded and unloaded with the extracts, were cut into circular disks of 6 mm diameter using a sterile cork borer. The disks were then placed on the inoculated agar plates, ensuring proper contact with the surface. Plates were incubated at 37°C for 24 h. After incubation, the diameter of the inhibition zones around each disk was measured in millimeters using a caliper. The experiment was performed in triplicate, and the mean inhibition zone diameter was calculated for each bacterial strain [31].

### 2.8. Determination of Minimum Inhibitory Concentration (MIC) and Minimum Bactericidal Concentration (MBC)

The MIC and MBC of the chitosan/Persian gum hydrogels loaded with Jaft and yarrow extracts were determined using a method adapted from the protocol described by Farasati Far et al. [32]. Hydrogels were freeze‐dried (lyophilized) to constant weight and then finely ground into a uniform powder using a sterile mortar and pestle, as recommended for solid‐state hydrogel materials. The lyophilized hydrogel powder was resuspended in sterile Mueller‐Hinton broth to prepare a stock suspension at a maximal concentration of 1000 mg/mL. The suspension was thoroughly vortexed to achieve an even dispersion of hydrogel particles, and two‐fold serial dilutions were performed to obtain concentrations ranging from 1000 to 7.81 mg/mL. For the MIC assay, 100 *μ*L of each hydrogel dilution was placed in a well of a sterile 96‐well microtiter plate, followed by 100 *μ*L of standardized bacterial suspension (1.5 × 10^6^ CFU/mL) of either *E. coli* or *S. aureus*, to yield a final volume of 200 *μ*L per well. Plates were incubated at 37°C for 24 h. The MIC was defined as the lowest concentration showing no visible turbidity compared with the growth control after incubation. Blank control wells containing hydrogel suspensions without bacteria were included in each plate to ensure that hydrogel particles did not contribute to visible turbidity. For the MBC, 20 *μ*L aliquots were drawn from wells showing no visible growth and plated onto Mueller‐Hinton agar. Plates were incubated at 37°C for 24 h. The MBC was defined as the lowest hydrogel concentration yielding no bacterial colonies. All tests were carried out in triplicate, and results are reported as mean ± SD [32].

### 2.9. Animal Study

#### 2.9.1. Study Design

A total of 30 mature female NMAR mice, weighing between 30 and 35 g, were obtained from the Center of Comparative and Experimental Medicine at Shiraz University of Medical Sciences. The mice were housed in standard cages with access to a standard rodent pellet chow diet (RoyanFeed, Isfahan, Iran) and tap water. Prior to the experiment, the animals were acclimatized to the new environment for 15 days at a temperature of 25^°^C ± 1^°^C, a 12‐h light/dark cycle, and a relative humidity of 40*%* ± 10*%* to minimize the impact of stress. During the acclimatization period, the mice were examined by a veterinarian to ensure their health. After the acclimatization period, the mice were randomly divided into three groups (*n* = 10 per group):

Group 1: control group (negative controls: untreated wounds and positive control: treated 1% sulfadiazine ointment).

Group 2: treated with the chitosan/Persian gum hydrogel alone.

Group 3: treated with the chitosan/Persian gum hydrogel loaded with yarrow and Jaft extracts.

The animals were housed individually in cages to prevent cross‐contamination between groups. All procedures were conducted in accordance with the guidelines for the care and use of laboratory animals and were approved by the Institutional Animal Care and Use Committee of Shiraz University of Medical Sciences [33].

#### 2.9.2. Wound Creation and Treatment

Two full‐thickness excisional wounds measuring 1 cm in diameter were created on the dorsal surface of each mouse under general anesthesia using ketamine (80 mg/kg) and xylazine (10 mg/kg). The wounds were left uncovered for 30 min to allow the formation of a fibrin clot. In Groups 2 and 3, the respective hydrogel formulations were applied to the wound beds, and the wounds were covered with sterile gauze and adhesive bandages. The dressings were changed every 3 days until complete wound closure was achieved [33].

#### 2.9.3. Wound Healing Assessment

The wound healing process was evaluated by measuring the wound area on Days 0, 3, 7, and 14 using a digital caliper and tracing wound margins onto transparent sheets. The percentage of wound closure was calculated relative to the initial wound area on Day 0.

Histological analysis using hematoxylin–eosin (H&E) staining was performed on tissue samples harvested on Days 3, 7, and 14. Paraffin‐embedded sections (5 *μ*m thickness) were prepared and stained. Assessment included qualitative evaluation of epithelialization, granulation tissue formation (including angiogenesis and collagen deposition), inflammation (cellular infiltrate), and the presence of skin appendages. Quantitative measurements were performed using ImageJ software (National Institutes of Health, United States) on calibrated digital micrographs. Epidermal thickness was measured at multiple points across the neoepidermis. Fibroblast numbers were quantified by counting spindle‐shaped cells within three distinct high‐power fields (400x magnification) in the granulation tissue area. Moreover, the existence of infection in wounds was also evaluated using the plate count method on Days 3, 7, and 14. In this context, the presence and extent of wound infection were evaluated using bacterial culture and colony counting. Wound swabs were collected on Days 3, 7, and 14, serially diluted in sterile saline, and plated onto Luria–Bertani (LB) agar plates. Plates were incubated at 37°C for 24–48 h, and colony‐forming units (CFU) per wound were calculated. Gross signs of infection (pus, excessive redness, and swelling) were also monitored during hydrogel dressing changes. Histological sections were also examined for bacterial aggregates and excessive inflammatory infiltrates. In this method, the hydrogel’s ability to prevent opportunistic wound infection under standard housing conditions was evaluated rather than employing a specific bacterial challenge model. Infection monitoring focused on quantifying the total bacterial load using CFU counts on non‐selective media (LB agar) and assessing inflammatory responses histologically [33].

### 2.10. Statistical Analysis

Data were analyzed using GraphPad Prism software (Version 6). One‐way ANOVA followed by Tukey’s post hoc test was used to compare the wound healing rates among the three groups. A *p* value less than 0.05 was considered statistically significant.

## 3. Results

### 3.1. Characterization of the Synthesized Hydrogel

The FTIR spectroscopy of the chitosan/Persian gum hydrogel revealed distinct absorption bands indicative of characteristic functional groups. In the pure hydrogel (Figure 2a), strong peaks corresponding to O‐H and N‐H stretching vibrations were observed around 3300 cm^-1^, while C‐H stretching was prominent near 2900 cm^-1^. The presence of amide I and II bands around 1650 cm^-1^ and 1550 cm^-1^, respectively, confirmed the presence of chitosan. For the hydrogel loaded with yarrow and Jaft extracts (Figure 2b), additional peaks were detected, which could be attributed to the bioactive compounds from the extracts, suggesting successful loading into the hydrogel matrix.

Figure 2(a) FTIR spectrum of chitosan/Persian gum hydrogel alone, highlighting characteristic absorption bands associated with functional groups. (b) FTIR spectrum of chitosan gel/Persian gum hydrogel loaded with Jaft and yarrow extracts, highlighting characteristic absorption bands indicative of successful incorporation of bioactive compounds.

**Figure:**
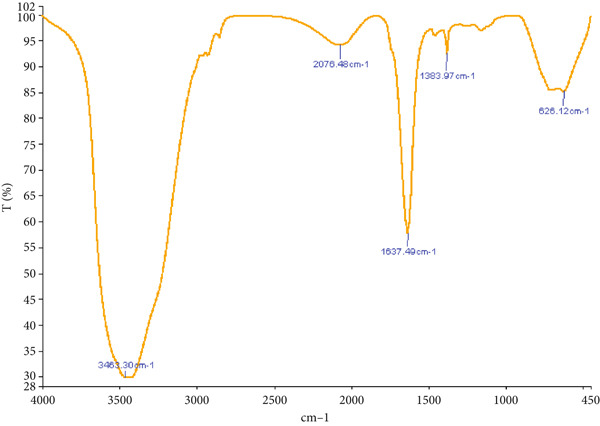
(a)

**Figure:**
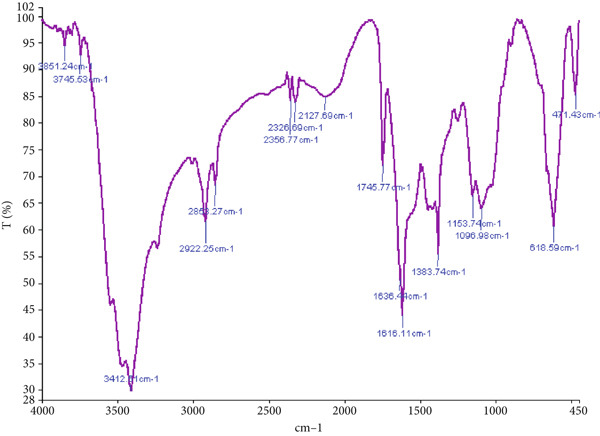
(b)

XRD analysis of the pure chitosan/Persian gum hydrogel (Figure 3) demonstrated characteristic diffraction peaks at 2*θ* values of approximately 23.54°, 30.99°, 36.79°, and 48.89°, corresponding to the crystalline structure of chitosan. The relative intensity of these peaks indicated a semicrystalline nature of the hydrogel, which is consistent with the partial crystallinity of chitosan and the amorphous nature of Persian gum.

Figure 3(a) XRD spectrum of chitosan gel/Persian gum, illustrating the crystalline structure and phase composition of the hydrogel matrix. (b) XRD spectrum of chitosan gel/Persian gum hydrogel loaded with Jaft and yarrow extracts, revealing the crystalline structure and phase composition of the hydrogel matrix after incorporation of the bioactive compounds.

**Figure:**
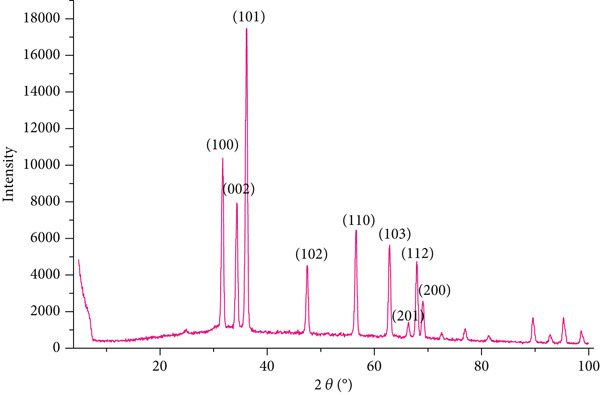
(a)

**Figure:**
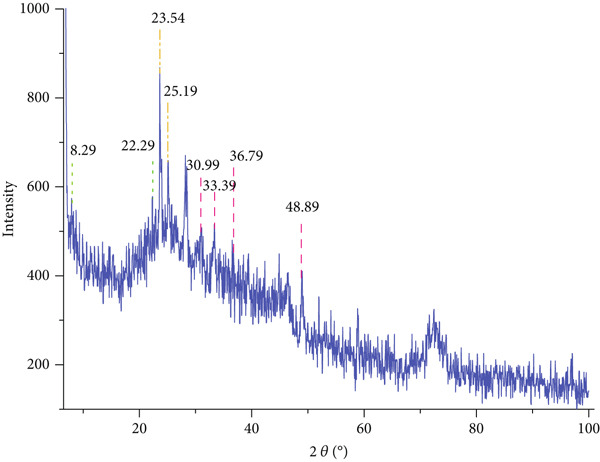
(b)

SEM images (Figure 4) provided insights into the morphology of the chitosan/Persian gum hydrogels loaded with oak and yarrow extracts. The SEM micrographs revealed a porous and interconnected network structure, which is typical for hydrogels. The size of the pores varied, and the inclusion of plant extracts appeared to slightly alter the surface morphology, indicating a successful integration of the extracts into the hydrogel matrix. The components’ sizes were measured, confirming that the hydrogel maintained a uniform structure conducive to potential applications in controlled release systems.

Figure 4(a) This SEM image shows the cross‐sectional morphology of the chitosan/Persian gum hydrogel loaded with Jaft and yarrow extracts. The structure is highly porous and layered, with interconnected pores visible throughout the matrix. Such morphology is favorable for fluid exchange, nutrient transport, and cellular infiltration, which are essential for wound healing applications. (b) This higher‐magnification SEM image highlights the fibrous network of the hydrogel matrix and the distribution of extract domains. Several extract‐loaded regions are annotated, with sizes ranging from approximately 410–3.75 *μ*m. These measurements provide a qualitative indication of the heterogeneity in extract particle size, but do not represent a full statistical analysis or histogram. The even distribution of extract domains suggests effective incorporation within the matrix.

**Figure:**
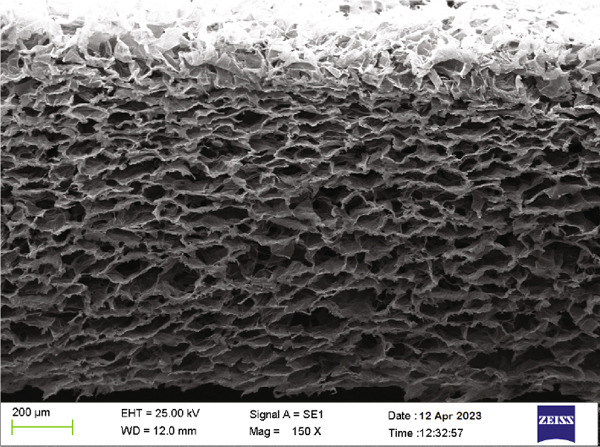
(a)

**Figure:**
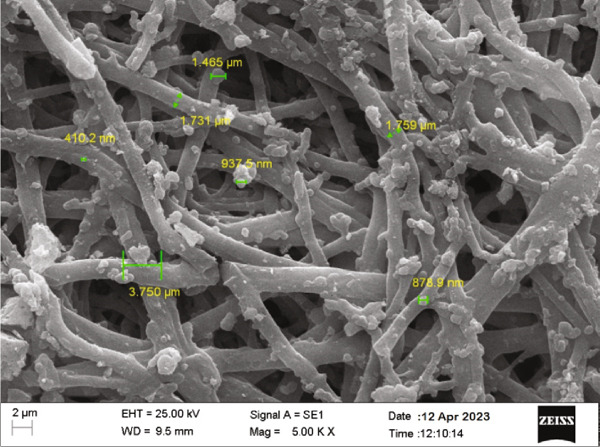
(b)

### 3.2. Antibacterial Activity of Synthesized Gel

The antibacterial activity of chitosan/Persian gum hydrogel, both with and without oak fruit peel and yarrow extracts, was assessed against *S. aureus* (gram‐positive) and *Escherichia coli* (gram‐negative). The chitosan/Persian gum gel without any extract exhibited negligible antibacterial effects against both bacterial strains. However, when loaded with oak and yarrow extracts, the hydrogel demonstrated significant antibacterial activity, effectively inhibiting the growth of both *S. aureus* and *E. coli*. Remarkably, the antibacterial performance of the extract‐loaded hydrogel was comparable to that of the positive controls, amoxicillin and tetracycline, used against *S. aureus* and *E. coli*, respectively. Notably, the hydrogel containing the extracts exhibited superior effectiveness against *E. coli*, surpassing even the conventional antibiotic treatment.

### 3.3. MIC and MBC Assessment

The antibacterial efficacy of the chitosan/Persian gum hydrogels loaded with oak (Jaft) and yarrow extracts was quantitatively assessed against *S. aureus* (Gram‐positive) and *E. coli* (Gram‐negative) using the broth microdilution method. All experiments were performed in triplicate, and results are presented as mean ± standard deviation (SD).

For *S. aureus*, the hydrogel exhibited a MIC of 12.75 ± 0.50 mg/L and a MBC of 25.5 ± 1.00 mg/L. In contrast, for *E. coli*, the hydrogel demonstrated significantly lower MIC and MBC values of 2.36 ± 0.12 mg/L and 4.72 ± 0.20 mg/L, respectively.

Statistical analysis was performed using one‐way analysis of variance (ANOVA) followed by Tukey’s post hoc test. The MIC and MBC values for *E. coli* were found to be significantly lower than those for *S. aureus* (*p* < 0.05), indicating a higher antibacterial potency of the hydrogel against Gram‐negative bacteria.

### 3.4. In Vitro Drug Release Study of Synthesized Hydrogel

**Figure 5:**
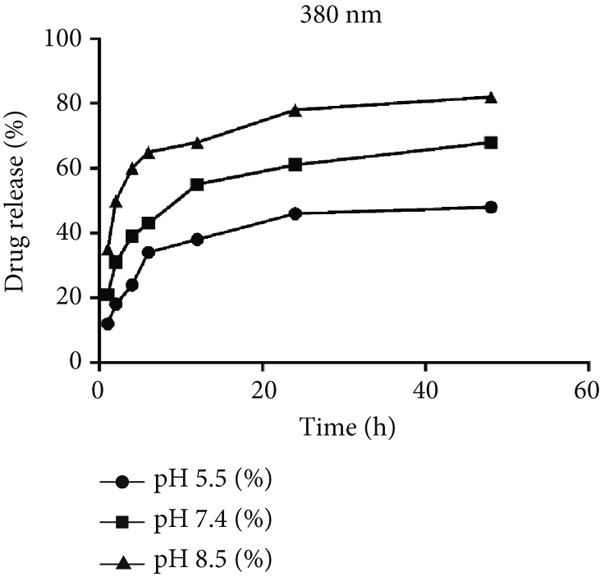
Release profile of Jaft and yarrow extracts from chitosan/Persian gum hydrogel in PBS at 37°C, demonstrating the cumulative release of bioactive compounds over time.

The in vitro drug release study of the synthesized hydrogel containing Jaft and yarrow extracts was conducted at two different pH levels, 5.5, 7.4, and 8.5 (Figure 5). The results indicated that at pH 5.5, the release of the extracts was moderated by strong charge interactions within the hydrogel matrix, involving electrostatic and hydrogen bonds due to the presence of hydroxyl and carboxyl groups. Conversely, at pH 7.4, the release rate was significantly higher, with 55 ± 2*%* of the extracts being released within the first 12 h. After 48 h, the release of extracts continued to be faster at pH 7.4 compared to pH 5.5. Moreover, at pH 8.5, we observed a markedly accelerated release profile of 78 ± 3*%* from loaded extracts that was liberated within 12 h (vs. 55 ± 2*%* at pH 7.4), reaching 82 ± 3*%* by 48 h. Statistical analysis using the chi‐square test confirmed that the release rate at pH 8.5 was significantly greater than at pH 7.4 and pH 5.5 at all examined time points (*p* < 0.05). Additionally, the ANOVA test demonstrated a significant increase in drug release over time at both pH levels (*p* < 0.05).

### 3.5. Evaluation of Wound Healing Efficacy of Chitosan Gel/Persian Gum–Loaded Extract

**Figure 6:**
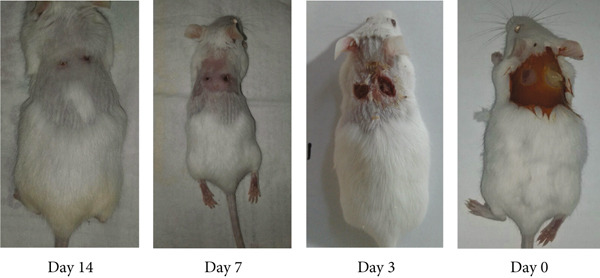
Wound healing process during Days 0–14 (left side wound positive control treated with sulfadiazine, right side wound treated with gel).

**Figure 7:**
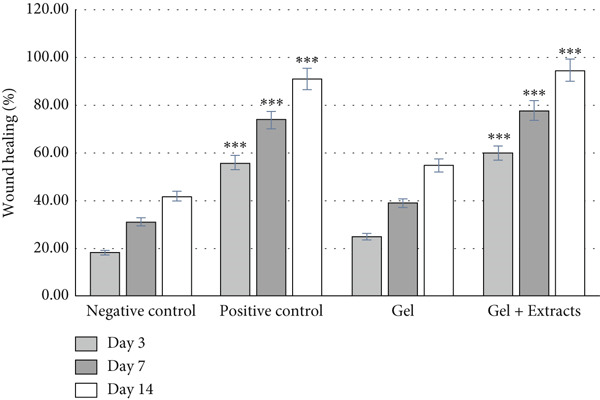
Percentage of wound healing during the 3rd, 7th, and 14th days after wound formation in the studied groups ( ^∗∗∗^ *p* < 0.001).

The macroscopic evaluation of wound sites was conducted on Days 0, 3, 7, and 14 postwounding (Figure 6). The group treated with chitosan gel/Persian gum containing extracts exhibited superior healing effects compared to the other groups, with no signs of infection or inflammation observed. In particular, the positive control group treated with sulfadiazine also demonstrated effective healing. The diameter of the wounds was measured, and the percentage of wound healing was calculated (Figure 7). The results indicated that the percentage of wound healing in the chitosan gel/Persian gum extract–treated group and the sulfadiazine group was significantly higher than in the negative control group (no treatment) and the group treated with chitosan gel/Persian gum without extract.

**Figure 8:**
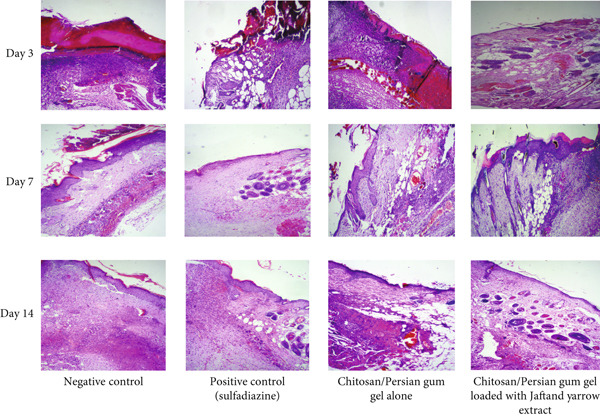
Hematoxylin–eosin staining of wound tissue on Day 14, demonstrating near‐complete epidermal regeneration and the presence of skin appendages in the positive control and extract‐treated groups, contrasted with incomplete regeneration and minimal inflammation in the negative control group.

Microscopic observations were conducted using H&E staining on Days 3, 7, and 14, with results depicted in Figure 8. On Day 3, all groups displayed some degree of inflammation and bleeding; however, the positive control and the group treated with the gel containing the extract exhibited reduced inflammation and bleeding compared to the other groups. By Day 7, histological examination revealed that the negative control samples showed incomplete epidermis regeneration, characterized by bleeding, fibrin clots, low collagen content, and significant inflammation. In contrast, the positive control and the group treated with the gel loaded with extracts demonstrated clear epidermal regeneration, increased angiogenesis, granulation tissue formation, and higher collagen fiber presence. On Day 14, the negative control group still displayed incomplete epidermis regeneration, with skin appendages absent, while the treated groups showed near‐complete epidermal regeneration, presence of skin appendages, and organized collagen fibers.

The thickness of the epidermis was evaluated on Day 14 (Figure 9a). The results indicated that the epidermal thickness in the positive control group receiving sulfadiazine and the group treated with the gel loaded with extracts was significantly greater than in the negative control groups treated with gel without extract.

Figure 9(a) Thickness of the epidermis in the studied groups after 14 days of treatment, showing significantly greater thickness in the sulfadiazine and extract‐loaded gel groups compared to the negative control groups ( ^∗∗∗^ *p* < 0.001). (b) Number of fibroblast cells in the studied groups after 14 days of treatment, showing significantly higher counts in the chitosan gel/Persian gum extract and positive control groups compared to the negative control groups ( ^∗∗^ *p* < 0.01). (c) Number of bacterial colonies observed in samples from the studied groups, indicating significantly lower colony counts in the sulfadiazine and chitosan gel/Persian gum loaded with Jaft and yarrow extract groups compared to the negative control groups ( ^∗∗∗^ *p* < 0.001).

**Figure:**
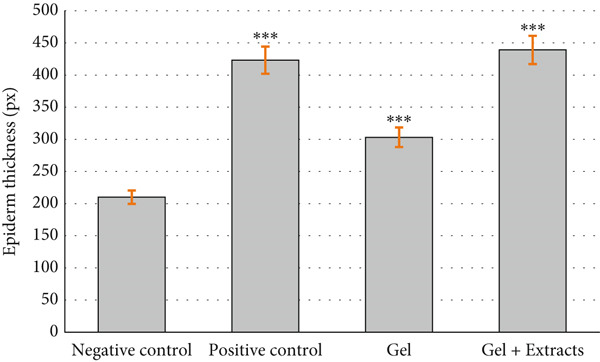
(a)

**Figure:**
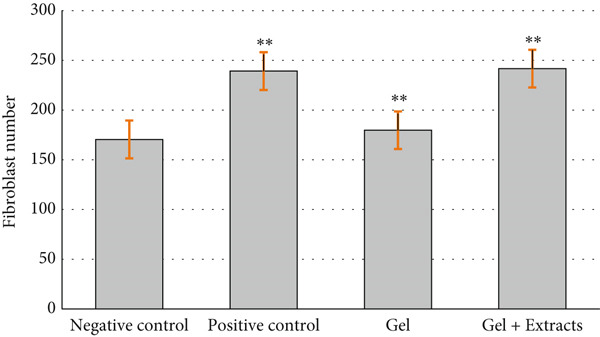
(b)

**Figure:**
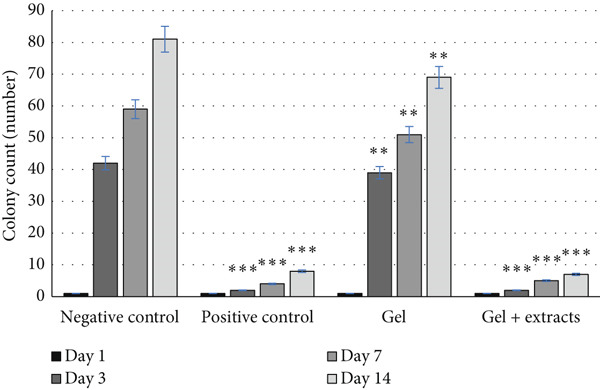
(c)

Fibroblast counts (Figure 9b) revealed that the groups treated with the chitosan gel/Persian gum containing extract and the positive control group had significantly higher fibroblast numbers after 14 days of treatment compared to the other groups. No significant difference was observed between the groups treated with chitosan gel/Persian gum without extract and the untreated group.

Infection evaluation was performed by assessing bacterial colonies on LB agar medium over the study period (Days 0, 3, 7, and 14). On Day 0, due to the disinfection of the wound environment, almost no colonies were observed. Over time, the number of colonies increased in all groups; however, the groups treated with sulfadiazine and the chitosan gel/Persian gum loaded with Jaft and yarrow extracts exhibited significantly fewer colonies compared to the negative control and the group treated with gel without extract, indicating a lack of infection in the former groups. The minimal colony formation in these groups suggests that the observed colonies were not indicative of infection, while infection was noted in the negative control and the group treated with gel without extract (Figure 9c).

## 4. Discussion

The skin, as a vital and multifunctional organ, plays an essential role in maintaining homeostasis by serving as a barrier against external threats, preventing dehydration, and facilitating sensory perception [2]. When the skin’s integrity is compromised, the body initiates a complex wound healing process consisting of several overlapping phases: hemostasis, inflammation, proliferation, and remodeling. Given the complexity of wound healing, selecting the appropriate wound dressing is paramount to effectively manage the process, accelerate wound closure, prevent infections, and promote tissue regeneration [2]. In this context, recent advances in biomaterials have led to the development of hydrogels as effective wound dressings due to their high moisture content, biocompatibility, and ability to incorporate bioactive compounds [34, 35]. This study focused on developing a chitosan/Persian gum hydrogel infused with Jaft and yarrow extracts, aiming to enhance wound healing through antimicrobial and anti‐inflammatory properties.

To validate the effectiveness of the hydrogel in promoting wound healing, a comprehensive analysis of its structural and compositional properties was conducted. In this regard, the successful synthesis of the chitosan/Persian gum hydrogel was confirmed through FTIR Spectroscopy, XRD, and SEM. The FTIR analysis revealed characteristic absorption bands corresponding to functional groups in both chitosan and Persian gum, indicating successful incorporation of these components into the hydrogel matrix. The additional peaks observed in the FTIR spectrum of the hydrogel loaded with Jaft and yarrow extracts suggested the presence of bioactive compounds from the extracts, aligning with findings from similar studies on polysaccharide‐based hydrogels [36, 37]. Moreover, XRD analysis demonstrated that the hydrogel possessed a semicrystalline structure, typical of chitosan‐based materials. The diffraction peaks observed were consistent with the crystalline nature of chitosan, while the amorphous nature of Persian gum contributed to the hydrogel’s flexibility and porosity [38, 39]. The semicrystalline structure is crucial for maintaining the mechanical integrity of the hydrogel while allowing for the controlled release of incorporated bioactive agents, as seen in other studies on similar materials [40, 41]. Finally, SEM micrographs revealed a porous and interconnected network structure, essential for facilitating the absorption and retention of fluids, which is critical for wound healing. The inclusion of Jaft and yarrow extracts slightly altered the surface morphology of the hydrogel, indicating successful integration of the extracts. This porous architecture is consistent with other chitosan‐based hydrogels, which have been shown to be effective in drug delivery and tissue engineering applications [42, 43].

In light of this characterization, the antimicrobial efficacy of the chitosan/Persian gum hydrogel was significantly enhanced by the incorporation of oak and yarrow extracts. The hydrogel exhibited strong antibacterial activity against both *S. aureus* (gram‐positive) and *E. coli* (gram‐negative), with the efficacy against *E. coli* surpassing that of conventional antibiotics used as positive controls. It is noteworthy that the chitosan/Persian gum hydrogel without plant extracts exhibited only weak antibacterial activity relative to the extract‐loaded formulation, despite the high total chitosan content. This observation may be attributed to the hydrogel’s dense, crosslinked matrix, which reduces the bioavailability of protonated amino groups on chitosan—key moieties responsible for bacterial interaction [44]. The presence of Persian gum, an anionic polysaccharide, may further neutralize chitosan’s cationic sites through electrostatic interactions, diminishing its antimicrobial effect [45]. Additionally, the crosslinking process and porosity of the hydrogel likely limited the diffusion of chitosan into the agar, affecting its ability to interact with bacterial cells in the disk diffusion assay. These findings underscore how formulation specifics, such as crosslinking density, polymer interactions, and matrix structure, can significantly modulate the antimicrobial performance of chitosan‐based hydrogels compared to chitosan in solution or as simple films [46]. This enhanced antibacterial activity can be attributed to the bioactive compounds present in Jaft and yarrow extracts, which include tannins, flavonoids, and phenolic acids. These compounds are known to disrupt microbial cell walls and inhibit essential enzymes, thereby exerting a potent antibacterial effect [2, 22, 24, 35, 47]. The bioactive compounds in Jaft and yarrow extracts contribute to wound healing through multiple complementary mechanisms. Tannins promote wound healing primarily through three pathways: scavenging of free radicals and reactive oxygen species (ROS), promoting wound contraction, and increasing the formation of capillary vessels and fibroblast proliferation [48]. This antioxidant activity is particularly beneficial in chronic wounds where oxidative stress impairs the healing process. Flavonoids, another crucial class of compounds in these extracts, enhance wound healing through their well‐established anti‐inflammatory, angiogenic, re‐epithelialization, and antioxidant effects [49]. Mechanistically, flavonoids act on various signaling pathways including Wnt/*β*‐catenin, TGF‐*β*, NF‐*κ*B, and PI3K/Akt, which regulate critical aspects of the wound healing cascade [49]. Phenolic acids present in the extracts accelerate wound contraction, reduce epithelialization period, protect against oxidative damage, and importantly, promote the secretion of growth factors such as VEGF and PDGF that are essential for tissue regeneration [50]. The combination of these compounds in our hydrogel formulation provides a synergistic effect that not only combats infection but also directly stimulates the cellular and molecular processes necessary for efficient wound healing. However, while this study successfully demonstrates the antimicrobial and wound healing capabilities of the chitosan/Persian gum hydrogel loaded with Jaft and yarrow extracts, antioxidant analyses were not conducted for this specific composite. Given the documented antioxidant properties of tannins, flavonoids, and phenolic acids in Jaft and yarrow extracts, the absence of such analyses limits a comprehensive evaluation of the hydrogel’s potential. Future studies should explore the antioxidant capacity of this formulation to fully characterize its role in mitigating oxidative stress during wound healing.

This connection between the enhanced antibacterial activity and the specific MIC and MBC values underscores the potential of the chitosan/Persian gum hydrogel as a robust alternative in fighting resistant bacterial infections. In this context, the MIC and MBC values determined for the hydrogel against *S. aureus* and *E. coli* were consistent with previous studies on chitosan‐based hydrogels loaded with natural antimicrobials [47, 51–53]. The lower MIC and MBC values observed for *E. coli* compared to *S. aureus* suggest that the hydrogel formulation is particularly effective against gram‐negative bacteria, which are typically more resistant to conventional antibiotics. This finding highlights the potential of the chitosan/Persian gum hydrogel as a powerful tool in combating bacterial infections, especially in cases where resistance to standard treatments is a concern [47].

Following these encouraging findings regarding antibacterial activity, the in vitro drug release studies conducted at pH 5.5 and 7.4 demonstrated the hydrogel’s pH‐responsive behavior, which is advantageous for targeted drug delivery in different physiological conditions. At pH 5.5, the release of bioactive compounds was moderated by strong charge interactions within the hydrogel matrix, resulting in a slower release rate. In contrast, at pH 7.4, the release rate was significantly higher, with 68% of the extracts being released within the first 12 h. This pH‐sensitive release profile is indicative of the hydrogel’s potential to deliver therapeutic agents in a controlled manner, making it particularly suitable for treating infected or inflamed tissues where the pH is typically lower than that of healthy tissues [54]. The ability to modulate drug release based on environmental pH is a critical feature for smart wound dressings, as it allows for the sustained and controlled release of bioactive compounds at the wound site [55]. This capability is particularly important in managing chronic wounds, where prolonged and localized drug delivery is essential for effective treatment [55]. The findings of this study are consistent with other research on pH‐responsive hydrogels, which have shown similar release profiles and potential for application in wound care and drug delivery systems [56–59]. To better mirror the alkaline conditions of chronic wounds, we extended our release assays to pH 8.5, observing significantly accelerated extract liberation of 68 ± 2*%* at 12 h and 82 ± 3*%* at 48 h, owing to enhanced polymer deprotonation and matrix swelling. These findings underscore the hydrogel’s smart, pH‐responsive performance across a broad physiological spectrum and suggest superior efficacy in chronic wound environments where elevated pH promotes accelerated drug delivery. While the incorporated Jaft and yarrow extracts are known for their anti‐inflammatory properties, as reported in prior studies, we note that these effects are inferred based on the hydrogel’s release profile and the established bioactivity of the extracts. The current study focused on characterizing the pH‐dependent release kinetics of the chitosan/Persian gum hydrogel, laying essential groundwork for its potential as a smart wound dressing. To fully validate its clinical applicability, future work will include comprehensive biocompatibility assessments, such as in vitro cytotoxicity assays with fibroblasts and keratinocytes and in vivo evaluations of inflammatory responses in animal models [56–59]. These planned studies will confirm the material’s safety and therapeutic potential, building on the promising release dynamics demonstrated here.

Following the successful demonstration of pH‐sensitive drug release, the focus shifted to evaluating the wound healing efficacy of the hydrogel. The wound healing efficacy of the chitosan/Persian gum hydrogel was evaluated through both macroscopic and microscopic assessments over a 14‐day period. The results showed that the hydrogel loaded with Jaft and yarrow extracts significantly enhanced wound closure compared to the untreated control group and the hydrogel without extracts. The rapid wound closure observed in the treated groups is indicative of the hydrogel’s ability to create a moist wound environment, promote cell proliferation, and reduce the risk of infection, all of which are critical factors in effective wound healing [10, 11, 28, 60].

Histological analysis further supported these findings, revealing enhanced epithelialization, increased fibroblast proliferation, and greater collagen deposition in the treated groups. By day 14, the treated wounds exhibited near‐complete regeneration of the epidermis, with organized collagen fibers and the presence of skin appendages, which are essential for restoring the normal function and appearance of the skin. In contrast, the untreated control group displayed incomplete regeneration, characterized by persistent inflammation, low collagen content, and the absence of skin appendages. These observations align with previous studies on chitosan‐based dressings, where the incorporation of bioactive compounds led to improved wound healing outcomes [1, 9, 11, 14, 15]. In this regard, the superior wound healing observed in the group treated with the chitosan/Persian gum hydrogel containing Jaft and yarrow extracts can be attributed to the combined antimicrobial, anti‐inflammatory, and regenerative properties of the hydrogel. The presence of bioactive plant extracts not only enhances the hydrogel’s ability to prevent infections but also promotes faster tissue repair and regeneration, making it a promising candidate for the development of advanced wound care products [2, 42].

These findings highlight the unique properties of the chitosan/Persian gum hydrogel, which not only accelerate wound healing but also differentiate it from other wound dressings in terms of therapeutic efficacy and clinical application. When compared in depth with commercial wound dressings, our chitosan/Persian gum hydrogel offers distinct advantages. Traditional wound dressings such as gauze and cotton wool lack the moisture‐retaining properties essential for optimal wound healing, while advanced dressings like hydrocolloids and alginates, though moisture‐retentive, often lack antimicrobial properties unless specifically impregnated with agents such as silver. Our hydrogel combines the beneficial properties of advanced dressings with natural antimicrobial and healing‐promoting compounds. Unlike silver‐containing dressings that may cause cellular toxicity at higher concentrations [48], our formulation utilizes natural extracts with minimal cytotoxicity. Compared to synthetic polymer‐based hydrogels that often employ chemical crosslinkers potentially harmful to tissues, our chitosan/Persian gum hydrogel utilizes natural polysaccharides with inherent biocompatibility. Synthetic antimicrobials, while effective in preventing wound infections, can contribute to antimicrobial resistance when overused or misused. In contrast, natural antimicrobials, such as the Jaft and yarrow extracts used in this study, may pose a lower risk of resistance development due to their complex, multitarget mechanisms of action. For instance, the tannins and flavonoids in these extracts disrupt microbial cell walls and inhibit enzymatic activity, offering a complementary approach to infection control [61]. Furthermore, many commercial formulations lack the pH‐responsive behavior demonstrated by our hydrogel, which enables intelligent drug delivery based on the wound environment [62]. The combination of natural polymers and plant extracts in our formulation represents a more sustainable and potentially cost‐effective alternative to petroleum‐derived synthetic dressings, addressing both environmental concerns and accessibility issues in resource‐limited settings.

While the current study did not explicitly evaluate long‐term stability or degradation kinetics, insights can be drawn from the hydrogel’s structural properties and existing literature on polysaccharide‐based systems. The semicrystalline structure observed in XRD analysis suggests potential stability under physiological conditions, as crystalline regions in chitosan are known to resist enzymatic degradation longer than amorphous domains [63]. However, natural hydrogels like this formulation often face challenges in maintaining structural integrity over extended periods due to hydration‐dependent softening [63, 64].

For clinical translation, the hydrogel’s pH‐responsive drug release mechanism aligns with the needs of chronic wound management, where prolonged and localized delivery of antimicrobials is critical [64]. The interconnected porous network observed via SEM may facilitate controlled degradation by allowing gradual infiltration of lysozymes, which cleave glycosidic bonds in chitosan [63]. While in vitro cytotoxicity assays demonstrated > 90% cell viability, long‐term in vivo biocompatibility studies would be necessary to assess immune response and degradation byproducts.

Future work should prioritize accelerated stability testing under varying humidity and temperature conditions to model real‐world storage. Lyophilization, a common preservation method for natural hydrogels [63], could enhance shelf life without compromising bioactivity. Scalability challenges, particularly in standardizing Persian gum purity and plant extract concentrations, must also be addressed to ensure batch‐to‐batch reproducibility—a known hurdle for natural polymer formulations [63, 64].

## 5. Conclusion

The study successfully developed and characterized a novel chitosan/Persian gum hydrogel loaded with oak and yarrow extracts, demonstrating its significant potential for enhancing wound healing. The hydrogel exhibited excellent biocompatibility, a semicrystalline structure, and a porous morphology, all of which are conducive to its application in wound management. The inclusion of Jaft and yarrow extracts significantly improved the hydrogel’s antibacterial properties, particularly against *E. coli*, and promoted faster wound closure compared to the untreated control and the hydrogel without extracts. The in vitro drug release studies confirmed the hydrogel’s pH‐responsive behavior, offering controlled and sustained release of bioactive compounds, which is advantageous for targeted drug delivery in different physiological conditions. The animal studies further validated the hydrogel’s efficacy, showing superior wound healing, enhanced epidermal thickness, increased fibroblast proliferation, and reduced infection rates in treated wounds. These findings suggest that the chitosan/Persian gum hydrogel, particularly when loaded with natural extracts, holds significant promise as a multifunctional wound dressing that can effectively promote tissue regeneration, control infections, and provide a conducive environment for healing. Future research could explore its long‐term effects in clinical settings and its potential applications in various types of wounds.

## Conflicts of Interest

The authors declare no conflicts of interest.

## Funding

This study was funded by the Shiraz University of Medical Sciences (10.13039/501100004320).

## Data Availability

Data are available upon request.
